# The associations between screen time-based sedentary behavior and depression: a systematic review and meta-analysis

**DOI:** 10.1186/s12889-019-7904-9

**Published:** 2019-11-14

**Authors:** Xiao Wang, Yuexuan Li, Haoliang Fan

**Affiliations:** 1 Department of Psychiatry, First Hospital of Shanxi Medical University, 85 Jiefang South Road, Taiyuan, 030001 Shanxi China; 2 Peking University Sixth Hospital (Institute of Mental Health), National Clinical Research Center for Mental Disorders & Key Laboratory of Mental Health, Ministry of Health (Peking University), Beijing, 100191 China; 3 Judicial Expertise Center of Qiongshan District, Haikou Municipal Public Security Bureau, Haikou, 570000 Hainan China; 4 Department of Forensic Science, Forensic Science Center of Hainan Medical University, Hainan Medical University, No. 3 Xueyuan Road, Longhua District, Haikou, 571199 Hainan China; 5 School of Forensic Medicine, Southern Medical University, Guangzhou, 510515 Guangdong China

**Keywords:** Sedentary behavior, Mental health, Depression, Odds ratio

## Abstract

**Background:**

The use of computers/TV has become increasingly common worldwide after entering the twenty-first century and depression represents a growing public health burden. Understanding the association between screen time-based sedentary behavior (ST-SB) and the risk of depression is important to the development of prevention and intervention strategies.

**Methods:**

We searched the electronic databases of Medline, Embase and the Cochrane Library. The odds ratio (OR) with corresponding 95% confidence intervals (CIs) was adopted as the pooled measurement. Subgroup analyses were investigated by stratified meta-analyses based on age, gender and reference group (reference category of screen time, e.g. 2 h/day, 4 h/day).

**Results:**

There were 12 cross-sectional studies and 7 longitudinal studies met the inclusion criteria. Overall, the pooled OR was 1.28 with high heterogeneity (I^2^ = 89%). Compared to those who reported less SB, persons reporting more SB had a significantly higher risk of depression. When the gender was stratified, the pooled OR was 1.18 in female groups while no significant association was observed in males. Among the 19 studies, 5 studies used a reference group with ST = 2 h/days (pooled OR = 1.46), 9 studies used ≥4 h as a reference group (pooled OR = 1.38), 2 studies used 1 h as a reference group (pooled OR = 1.07) and for the remaining 3 studies, hours of ST were calculated as a continuous variable (pooled OR = 1.04).

**Conclusions:**

ST-SB is associated with depression risk and the effects vary in different populations. In addition, valid objective measures of SB should be developed in future studies.

## Background

The use of computers/TV has become increasingly common worldwide after entering the twenty-first century [1], and there has been a large increase in the number of workers whose major job is computer-related [2, 3]. Moreover, both adolescents and adults also spent a large amount of time on the computer or smartphone or watching television. With advances in technology, screen time (ST), including watching television, using a computer and playing video games, is becoming a central component of the daily lives [4] and the most common sedentary behavior [5] (i.e., activities that require minimal body movement resulting in low energy expenditure similar to that at resting level [1.0 to 1.5 metabolic equivalents (METs)] [6]). Previous studies have shown that screen time-based sedentary behavior (ST-SB) is associated with increased risk for a variety of physical diseases, such as cardiovascular disease [7], obesity [8], and diabetes [9]. Moreover, ST-SB also influences mental health, such as sleep problems [10], anxiety disorders [11] and depression [12].

Currently, mental disorders are widely recognized as a major contributor (14%) to the global burden of disease, and depression is one of the most prevalent mental disorders [13]. Indeed, the World Health Organization (WHO) ranked major depression as one of the most burdensome diseases in the world [14]. Major depression has increased from the 15th-leading cause of adult disease burden in 2000 to the 11th-leading cause in 2010 [15]. According to new estimates of depression released by the WHO, the number of people living with depression increased by 18% between 2005 and 2015. Depressive disorders are ranked as the single largest contributor to nonfatal health loss (7.5% of all years lived with disability). The prevalence varies across the world, from a low incidence of 2.6% among males in the Western Pacific Region to 5.9% among females in the African Region. Furthermore, depressive disorders are projected to be the second leading cause of disease burden worldwide by 2030 and are the leading cause in high-income countries [16]. In addition, the onset of depression is common in adolescents and young adults [17–19], who may spend much more time on computers than older persons, coinciding with a pivotal period of physical and psychological development, and can lead to poorer psychosocial functioning, lower life and career satisfaction, more interpersonal difficulty, higher need for social support, more comorbid psychiatric conditions, and increased risk of suicide.

The median age of onset (50th percentile on the age-of-onset distribution) was approximately 30 for major depressive disorders [20]. Currently, many adults study or work in front of computers, and ST-SB has become a common and important issue not only for adolescents but also for adults. Therefore, understanding the association between ST-SB and the risk of depression among adults is also important to the development of prevention and intervention strategies. Many studies have investigated the association among different populations; however, the results were inconsistent. Some studies showed that longer ST might lead to a higher prevalence of depressive-related problems, while some studies thought this association was not significant. Thus, this systematic review was conducted to explore whether ST-SB influenced the risk of depression.

## Methods

### Literature search strategy

A structured electronic search of publications from 2000 to 2018 was conducted, since the 2000’s saw an increase in sedentary behavior levels in the population with the widespread use of online technology [18]. Databases included Medline, Embase and the Cochrane Library. The following search strings were used: (depression OR depressive OR dysthymia OR mental health OR mental illness OR Psychinfo) AND (sedentary behav* OR sitting OR TV OR television OR computer OR screen OR smartphones OR tablets OR iPads). These strings were further limited to peer-reviewed publications written in English. First, titles and abstracts of articles identified in the search process were assessed for suitability. Second, the studies listed in the references of the articles were reviewed. The retrieval was conducted in Feb 2019. The full texts of the studies that met our criteria were downloaded after primary selection by reading the titles and abstracts.

### Study selection criteria

The risk of depression was defined as either diagnosed depression disorders (including major depressive disorder, dysthymic disorder and depressive disorder not otherwise specified) or the likelihood of developing or experiencing nonclinical depressive symptoms. Studies were considered eligible if they: (1) were observational studies, including cohort, case-control, and cross-sectional studies; (2) examined the risk of depression specifically; (3) assessed screen-time-based sedentary behavior; (4) concluded OR and 95% CI/se/*p* values; and (5) included participants aged 18 years or over.

### Data extraction

The following study characteristics of the identified studies were extracted: the first author, year of publication, country of origin, size of study population, study design, sample size, age, measures used of depression and ST-SB, analysis method and study results in terms of the association between ST-SB and risk of depression.

### Quality assessment

A modified version of an eight-component rating scale [21] was used to evaluate the methodological quality of the included studies. Because only observational studies were included in this review, six methodological components were included in the modified version: selection bias (e.g., response rate, representativeness), study design (e.g., cross-sectional, cohort, etc.), confounders (e.g., controlling for age, socioeconomic position, etc.), data collection methods (e.g., valid, reliable), withdrawals and dropouts (e.g., percent providing full data) and analyses (e.g., appropriateness of study design). Each of the components was given an overall section rating (weak, moderate, or strong). If one of these components was not described in the study included, for example, it said ‘more detail was described elsewhere’, we would try to find other papers that used the same database to provide this information. After all components were rated, a global rating for this paper of weak (if ≥2 of the components were scored weak), moderate (if < 3 components were scored strong with no more than one weak score), or strong (if ≥3 components were scored strong and ≤ 1 component was scored weak) was given to each study. Two reviewers (Wang and Li) independently assessed the methodological quality of these studies. Scoring discrepancies were resolved via consensus.

### Statistical analyses

The odds ratio (OR) with corresponding 95% confidence intervals (CIs) was used as a measurement to evaluate the association between ST-SB and depression. Adjusted effect sizes were used if available. Reports stratified by gender were treated as separate reports. Finally, because most of the studies included in this meta-analysis were not functionally identical, a DerSimonian and Laird random effects model was used to attain an overall OR and 95% CI. The combined effect size was evaluated using the inverse variance method. Heterogeneity between studies was tested using Cochran’s χ2 statistic and the *I*^*2*^ statistic. Generally, an *I*^*2*^ value of < 25%, corresponds to low heterogeneity, a value of 25–50% corresponds to moderate heterogeneity, and a value > 50% corresponds to strong heterogeneity between studies. Publication bias was assessed using funnel plots. Subgroup analyses were used to identify sources of heterogeneity. Based on the literature, the prevalence of depression differs by gender [22, 23] and age [24, 25]. In addition, the reference group (reference category of screen time, e.g., 2 h/day, 4 h/day) and study design also influenced ORs. Therefore, subgroup analyses were investigated by stratified meta-analyses based on age, gender, reference group, and study design. When an individual study reported effect sizes by gender, it would be divided into two parts in the subgroup analyses of gender. All *P* values were two-sided analyses, and 0.05 was considered statistically significant. All these analyses were conducted using R5.3 software (meta package and metagen package).

## Results

### Characteristics of the included studies

**Fig. 1:**
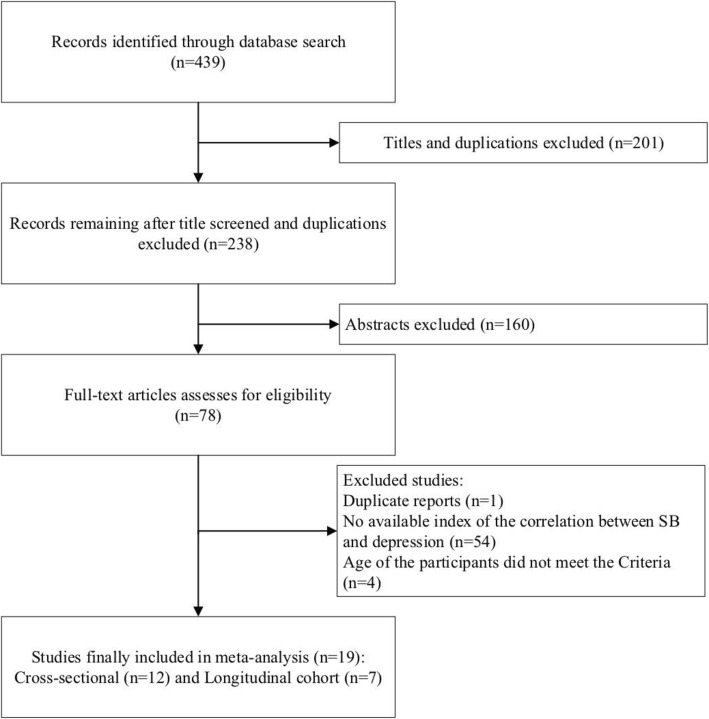
Flowchart of the article screening process

Our literature search yielded 439 studies (see Fig. 1). A total of 238 studies were screened by title. After a further screening of abstracts (*n* = 160) and full papers (*n* = 78), a total of 19 studies were included in the review. There were 12 cross-sectional studies and 7 longitudinal studies that met the inclusion criteria, including a total of 232,581 participants (118,991 in cross-sectional studies and 113,590 in longitudinal studies). The characteristics of the included studies are summarized in Table 1, including the author, year of publication, country, type of study, sample size, mean age, measures of depression and ST-SB, and quality scoring. The sample sizes ranged from 397 to 49,821. Fifteen studies involved both male and female participants, while 4 studies [29, 31, 33, 39] involved only female participants. Among these 15 studies, gender groups were analyzed separately in 2 studies. Several reference categories were used in the 19 analyzed studies. Nine studies used 4 h/day or over (cumulative) as the reference category, five used 2 h/day (cumulative), two used 1 h/day and three analyzed continuous ST. The risk of depression (depression symptoms or depression disorders) was measured using various measures, including the General Health Questionnaire (GHQ-12), Centers for Epidemiologic Studies–Depression Scale (CES-D), Patient Health Questionnaire (PHQ), Self-rating Depression Scale (SDS), Self-reported symptoms of depression, World Mental Health Composite International Diagnostic Interview (WMHCIDI), clinically diagnosed depression, and Edinburgh Postnatal Depression Scale (EPDS) (Table 1). For more details, see Additional file 1.

**Table 1:** Characteristics of the included studies

Paper	Country	Study design	Sample size	Age	Depression indicator	Sedentary behavior indicator	Reference categories	Methodological quality score
Primack et al.2009 [26]	USA	Cohort	4142	Mean(SD) age at follow-up: 21.8(1.8) years old	CES-D (20-item)	Self-report hours of exposure to electronic media	Continuous	Strong
Teychenne et al. 2010 [27]	Australia	Cross-sectional	3645	18–45 years old	CES-D (10-item)	Self-reported sitting time	1 h	Strong
Vallance et al.2010 [28]	Australia	Cross-sectional	2862	Mean(SD) age: 45.7(13.7) years old	PHQ-9	ActiGraph AM-7164 accelerometer	> 4 h	Strong
Lucas et al. 2011 [29]	USA	Cohort	49,821	30–55 years old	Clinical depression	Self-reported sitting time	1 h	Weak
Thomée et al. 2012 [30]	Sweden	Cohort	4163	20–24 years old	Self-reported symptoms of depression	Self-report computer time	2 h	Strong
Breland et al. 2013 [31]	USA	Cross-sectional	535	18–96 years old	PHQ-8	Self-reported screen time	> 4 h	Weak
Sloan et al. 2013 [32]	Singapore	Cross-sectional	4337	18–79 years old	GHQ-12	GPAQ v2	2 h	Strong
Van et al.2013 [33]	Australia	Cohort	8950	50–55 years old	CES-D (10-item)	Self-reported sitting time	> 4 h	Moderate
Arredondo et al.2013 [34]	USA	Cross-sectional	397	43.4 ± 16.9 years old	PHQ-9	GPAQ	Continuous	Strong
Feng et al. 2014 [35]	China	Cross-sectional	1106	18.9 ± 0.9 years old	SDS	Self-reported sitting time	2 h	Strong
Wu et al. 2015 [36]	China	Cross-sectional	4747	Mean(SD) age: 19.26(1.40) years old	CES-D	Self-reported screen time	2 h	Strong
Sui et al. 2015 [37]	China	Cohort	4802	18–80 years old	CES-D (10-item)	Self-reported TV or riding in a car time	Continuous	Moderate
Wu et al. 2016 [38]	China	Cross-sectional	2521	Mean(SD) age: 18.43(0.96) years old	CES-D (20-item)	Self-reported screen time	2 h	Strong
Padmapriya et al. 2016 [39]	Singapore	Cohort	1144	30.7 ± 5.1 years old	EPDS	Self-reported sitting time	> 4 h	Strong
Madhav et al.2017 [40]	USA	Cross-sectional	3201	20–74 years old	PHQ-9	Self-reported TV or computer time	> 4 h	Strong
Barros et al.2017 [41]	Brazil	Cross-sectional	49,025	18–59 years old	PHQ-9	Self-reported TV time	> 4 h	Strong
Nam et al.2017 [42]	South Korea	Cross-sectional	4145	20 years old and over	PHQ-9	Self-reported sitting time	> 4 h	Strong
Hallgren et al. 2018 [43]	Sweden	Cohort	40,569	Mean(SD) age: 51.6(16.1) years old	Clinical diagnosis	Self-reported screen time	> 4 h	Moderate
Stubbs et al. 2018 [44]	China, Ghana, India, Mexico, Russia, and South Africa	Cross-sectional	42,469	Mean(SD) age: 43.8(14.4)years old	WMHCIDI	Self-reported screen time	> 4 h	Strong

### Methodological quality

Methodological quality scores are provided in Additional file 2. We classified the overall quality of evidence (strong, moderate and weak) based on the modified version of an eight-component rating scale. Three longitudinal studies demonstrated a moderate methodological quality, and two studies (one cross-sectional, one longitudinal) received a weak methodological quality rating.

### ST-SB and depression risk

**Fig. 2:**
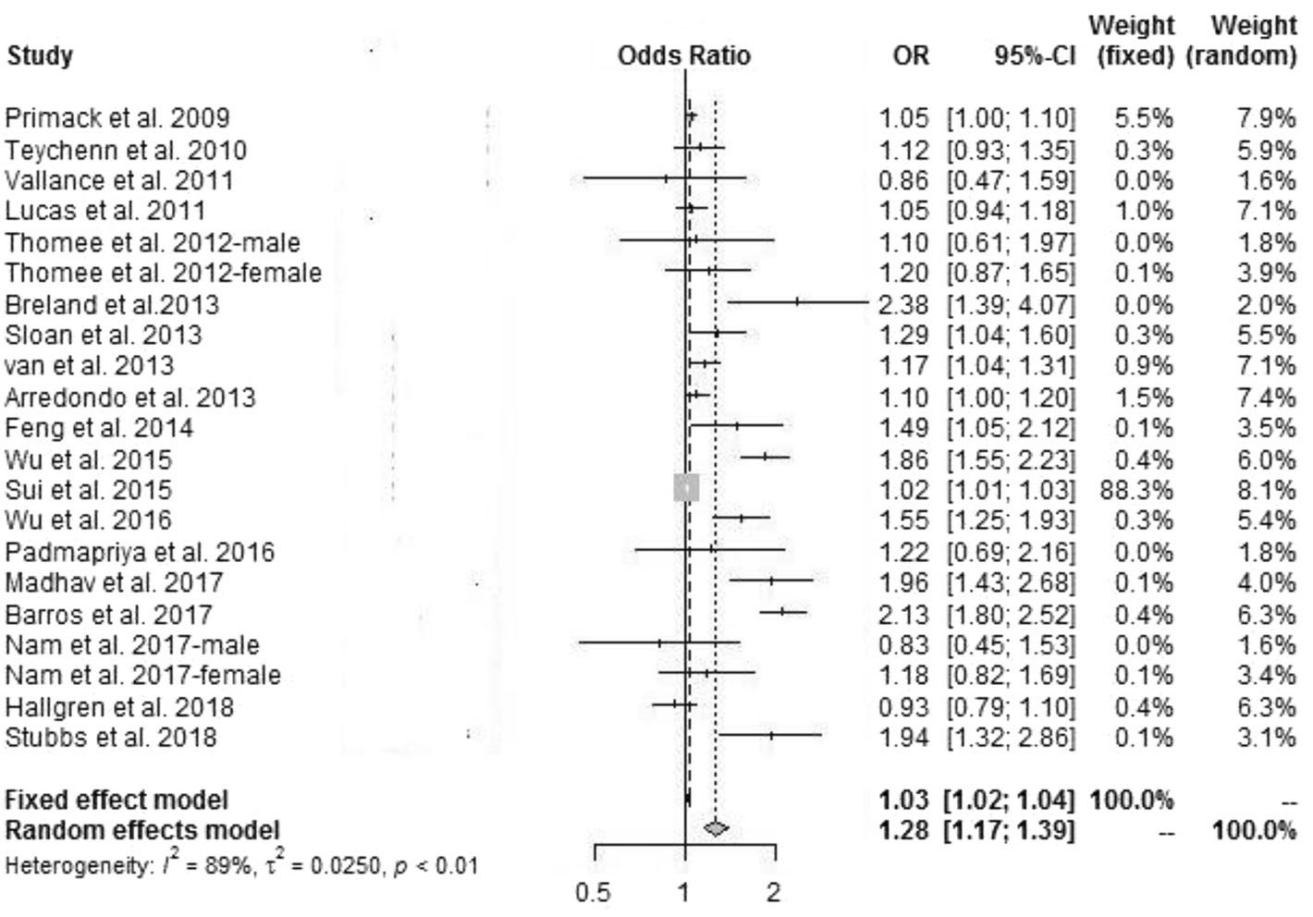
Forest plot of the association between depression risk and ST-SB

To analyze the association between ST-SB and depression, we used a random effect model to calculate the total OR and analyze the heterogeneity. As presented in Fig. 1, the overall pooled OR was 1.28 (95% CI 1.17 to 1.39; *p* < 0.01) with high heterogeneity (I^2^ = 89%) (Fig. 2). Persons reporting more SB had a significantly higher risk of depression than those who reported less SB.

To find the potential sources of heterogeneity, we conducted a group of subgroups analysis of gender, age, reference group and study design. When the gender was stratified, in female groups, the pooled OR was 1.18 (95% CI 1.03 to 1.35; *p* = 0.09) with moderate heterogeneity (I^2^ = 48%), and in male groups, the pooled OR was 0.96 (95% CI 0.63 to 1.47; *p* = 0.51) with low heterogeneity (I^2^ = 0%). No significant associations were observed in males. However, in studies that did not consider gender, the pooled OR was 1.32 (95% CI 1.18 to 1.48; *p* < 0.01) with high heterogeneity (I^2^ = 93%) (Additional file 3: Figure S1). In addition, when the age was presented into 2 groups (young adults and all adults), in the young adult groups, the pooled OR was 1.36 (95% CI 1.05 to 1.77; *p* < 0.01) with high heterogeneity (I^2^ = 90%), and in the all adults groups, the pooled OR was 1.25 (95% CI 1.11 to 1.41; p < 0.01) with high heterogeneity (I^2^ = 89%) (Additional file 3: Figure S2). To take the reference group into consideration, 5 studies used a reference group with ST = 2 h/days, and the pooled OR was 1.46 (95% CI 1.25 to 1.71; *p* = 0.06) with high heterogeneity (I^2^ = 52%); 9 studies used ≥4 h as a reference group, and the pooled OR was 1.38 (95% CI 1.08 to 1.77; p < 0.01) with high heterogeneity (I^2^ = 88%). Two studies used 1 h as a reference group, and the pooled OR was 1.07 (95% CI 0.97 to 1.18; *p* = 0.57) with low heterogeneity (I^2^ = 0%). For the remaining 3 studies, ST was calculated as a continuous variable, and the pooled OR was 1.04 (95% CI 1.00 to 1.08; *p* = 0.12) with high heterogeneity (I2 = 54%) (Additional file 3: Figure S3). Finally, to take the study design into consideration, 7 studies were cohort studies, and the pooled OR was 1.02 (95% CI 1.01 to 1.03; *p* = 0.41) with low heterogeneity (I^2^ = 3%), while the remaining 12 studies were cross-sectional studies, and the pooled OR was 1.48 (95% CI 1.25 to 1.74; *p* < 0.01) with high heterogeneity (I^2^ = 82%) (Additional file 3: Figure S4).

### Publication bias analysis

Begg’s rank correlation test (*p* = 0.5459) was conducted for publication bias evaluation. The result indicated that no significant publication bias existed in the meta-analysis. The above results indicated that the conclusions of our study were stable and credible (see Additional file 4: Figure S5).

## Discussion

This study aimed to investigate the association between ST-SB and depression with a meta-analysis, as previous studies showed inconsistent results. The results of the meta-analysis showed that most of the subjects with more than 2 h/d ST-SB were more likely to have depression. When ST was considered as a continuous variable, the associations between ST and depression became small yet remained statistically significant. Some mechanisms may explain the relationship between SB and the risk of depression. First, long-term SB might give rise to biological pathway disturbances including central nervous system arousal or sleep disturbances [45, 46]. Second, physical activity has been shown to be beneficial for reducing depressive symptoms [47]. However, some studies showed that even when controlling for physical activity and other demographic variables, the populations that reported high levels of screen time were more likely to be depressed than those who did not, suggesting that the effects of screen time are independent of physical activity [31]. Another explanation refers to social interaction: prolonged sedentary behaviors, such as television viewing, may lead to social solitude and withdrawal from interpersonal relationships, which have been linked to increased feelings of social anxiety [48]. Furthermore, these studies also showed a positive association between SB and obesity, which is explained by the mechanism through which SB is associated with energy-dense snack consumption and snacking behavior [49], and depression has been shown to be associated with obesity [50, 51].

In addition, according to the results of the subgroup analysis, there were significant differences between these associations in females and males. In the female population, the association was significant, while in the male population, it was not. This might be because of the increasing prevalence of mental health problems among females [52]. Furthermore, men and women use different coping mechanisms when dealing with depression. Women are more likely to internalize and ruminate on their condition, whereas men are more likely to engage in externalizing or distracting activities [53]. Thus, when screen time increases, females would likely have less time to communicate with others and would become more introverted, whereas males may shift their attention to other affairs. Thus, excessive time devoted to media may affect female users more substantially [54]. Moreover, using different reference categories led to different results. There was a week association between SB and depression risk in studies using 0–1 h/day as the reference category, while the association became stronger when using 2 h/day or more as the reference category. This finding provides better clarification of the association between ST-SB and depression risk, indicating that ST in moderation may not be associated with higher levels of depression. One hypothesis was that there was a curvilinear dose-response association between ST and the risk of depression. Some guidelines and recommendations [55] emphasized an overall positive association between ST-SB and morbidity risk. However, studies have shown that when ST is limited to 0–2 h/day, ST-SB is associated with a lower risk of depression, and the lowest risk is detected at ST of 1 h/day [4]. The selection of reference categories should be considered in future studies on SB. The results of the subgroup study by study design showed a consistent association in cohort and cross-sectional studies, but the heterogeneities were different, potentially because of the methodological limitations of cross-sectional studies. To demonstrate the association, cohort studies could provide higher grade evidence than cross-sectional studies [56].

Some caveats must be discussed. As the heterogeneity was quite high (approximately 90%), the factors that mainly explained this heterogeneity must be explored. Based on the results of the subgroup analysis, we found that gender, reference group and study design influenced the heterogeneity of the overall meta-analysis. In addition, distinct from chronic diseases such as hypertension, which could be diagnosed by objective indicators, information about depression disorders or depressive symptoms was often collected according to self-reported respondent answers to questions. The fieldwork of different studies was carried out by different interviewers, and the diagnoses could vary even though the instruments were the same. Moreover, there were several limitations to this review. First, most studies employed a cross-sectional study design, so these studies were limited by several methodological weaknesses. The cross-sectional character of these studies does not allow causal inferences to be made because relationships were unable to be determined. Second, SB was measured using retrospective self-report measures in most of the studies, which is subject to recall bias. In addition, mental health was possibly underestimated by respondents because of the stigma associated with psychological questions. Third, uncontrolled variables may have influenced the results. In this review, some studies controlled only social demographic variables such as age and gender, while physical activity and weight were also included as covariates in some studies. Further studies with proper controls for relevant covariates are needed to clarify this issue.

In future studies, valid objective measures of sedentary behavior are needed. Not only the dose (e.g., frequency, duration) but also the context (e.g., TV viewing, computer use, smartphone use) should be included in a structured or semistructured questionnaire. Additionally, some objective measures of sedentary behavior (e.g., accelerometers and posture monitors) are recommended. Moreover, some studies have focused on the linear or nonlinear relationships between ST-SB and depression [57, 58]. Further studies should be carried out to estimate the dose-response relationship between ST-SB and depression, exploring the appropriate time limit for ST-SB.

## Conclusion

ST-SB is associated with a higher risk of depression, especially when it exceeds 2 h/day. In the female population, the association between SB and risk of depression is significant, while in the male population, no significant associations were observed. Our review supports the current recommendations of limiting ST to promote mental health, especially in women. In addition, valid objective measures of sedentary behavior should be developed in future studies to explore appropriate time limits for ST-SB.

## Supplementary information

**Additional file 1.** Detailed characteristics of the included studies.

**Additional file 2.** Methodological quality assessment checklist for observational studies.

**Additional file 3.** Forest plot of subgroup analyses the association between depression risk and ST-SB.

**Additional file 4.** Test for Publication Bias.

## Data Availability

The materials used in the present study is available by request from all academic based researchers by a contact to the corresponding author.

## Abbreviations

CI: Confidence Interval
OR: Odds Ratio
SB: Sedentary Behavior
ST: Screen Time
ST-SB: screen time-based sedentary behavior
YLD: Years Lived with Disability
