# Supplementary material for: The associations between screen time-based sedentary behavior and depression: a systematic review and meta-analysis
Source: BMC Public Health. 2019 Nov 14;19:1524. doi: 10.1186/s12889-019-7904-9 (PMC6857327; doi:10.1186/s12889-019-7904-9)
Supplement: Supplementary file 3 — Additional file 3. Forest plot of subgroup analyses the association between depression risk and ST-SB. [file 12889_2019_7904_MOESM3_ESM.docx]

Forest plot of subgroup analyses the association between depression risk and ST-SB:


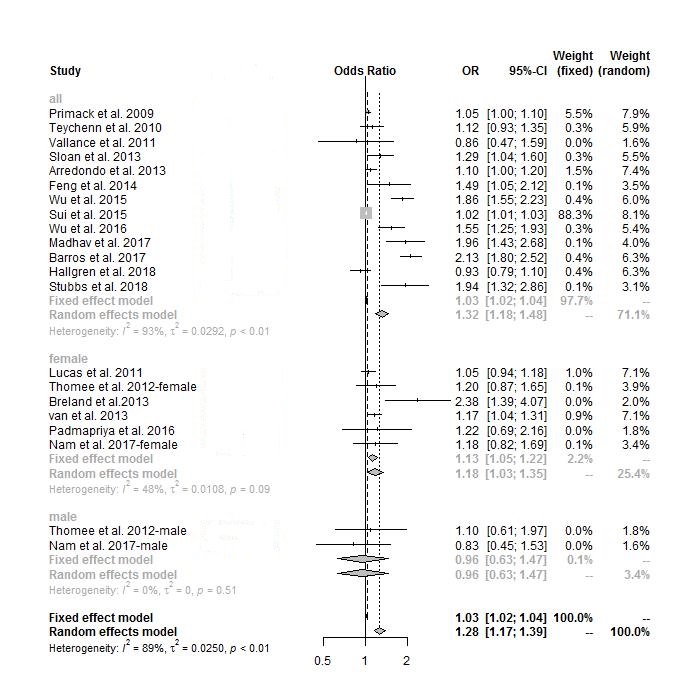


Figure 1. Forest plot of subgroup analyses the association between depression risk and ST-SB (by gender).


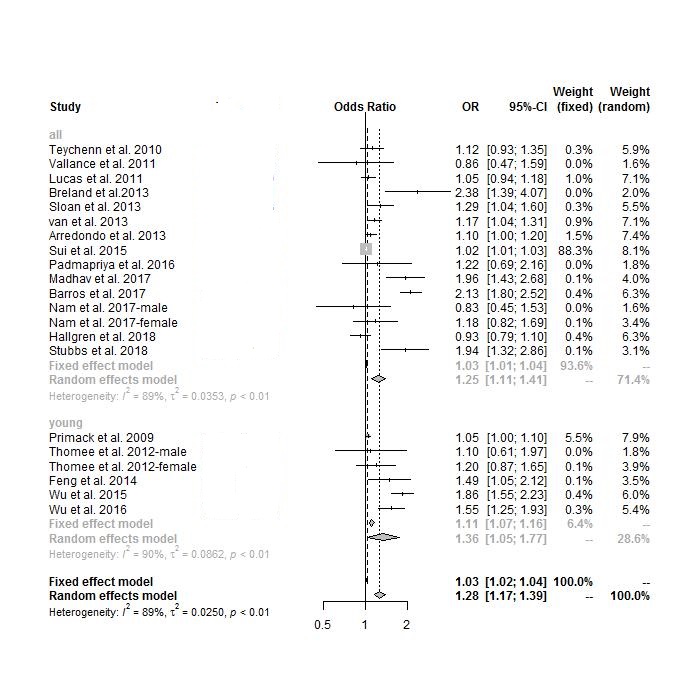


Figure 2. Forest plot of subgroup analyses the association between depression risk and ST-SB (by age).


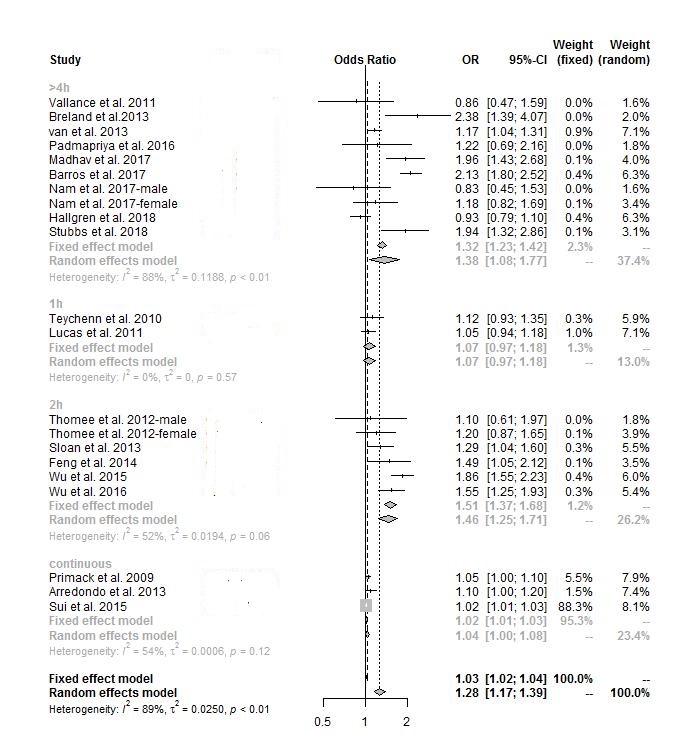


Figure 3. Forest plot of subgroup analyses the association between depression risk and ST-SB (by reference groups).


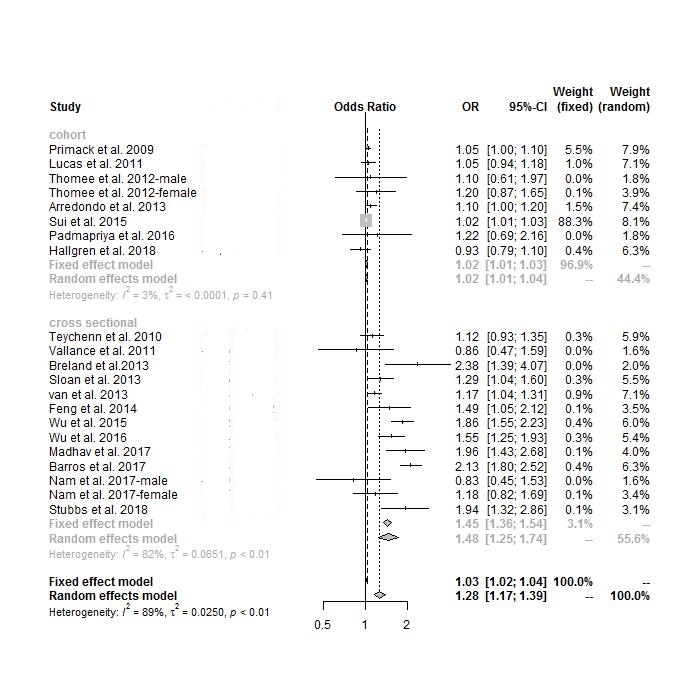


Figure 4. Forest plot of subgroup analyses the association between depression risk and ST-SB (by study design).
