# Supplementary material for: The associations between screen time-based sedentary behavior and depression: a systematic review and meta-analysis
Source: BMC Public Health. 2019 Nov 14;19:1524. doi: 10.1186/s12889-019-7904-9 (PMC6857327; doi:10.1186/s12889-019-7904-9)
Supplement: Supplementary file 4 — Additional file 4. Test for Publication Bias. [file 12889_2019_7904_MOESM4_ESM.docx]

Test for Publication Bias

Begg’s Test

adj. Kendall’s Score (P-Q) = -20

Std. Dev. Of Score = 33.12

Number of studies = 19

Z = -0.60

p = 0.5459


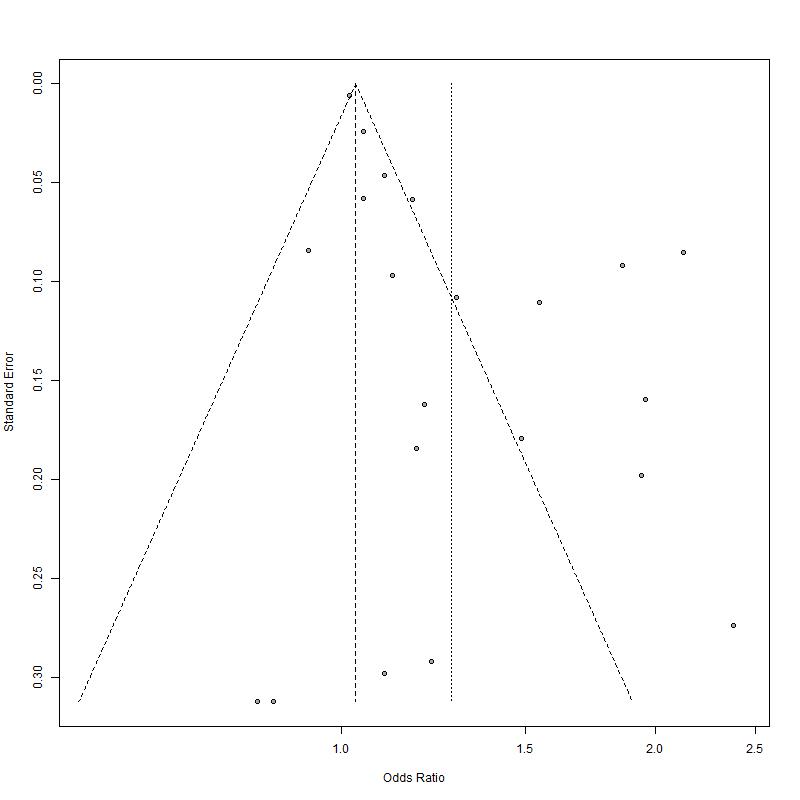


Figure S5. Funnel plot of publication bias.
